# Pharmacological manipulation of GABA-driven activity *in ovo *disrupts the development of dendritic morphology but not the maturation of spinal cord network activity

**DOI:** 10.1186/1749-8104-5-11

**Published:** 2010-04-08

**Authors:** Yone J Yoon, Alexander P Gokin, Miguel Martin-Caraballo

**Affiliations:** 1 Department of Biology, University of Vermont, Burlington, VT 05405, USA

## Abstract

**Background:**

In the adult nervous system, GABA acts as a major inhibitory neurotransmitter; however, at early stages of neurodevelopment, GABA receptor activation leads to membrane depolarization and accumulation of [Ca^2+^]_i_. The role of excitatory GABAergic neurotransmission in the development of the nervous system is not fully understood. In this study, we investigated the role of excitatory GABA-driven activity in regulating the dendritic morphology and network function in the developing chicken spinal cord.

**Results:**

Both bicuculline, a GABA receptor antagonist, and muscimol, a GABA agonist, inhibit the generation of spontaneous network activity in the isolated spinal cord at E8 or E10, indicating that altering GABA receptor activation disrupts the generation of spontaneous network activity in the chicken spinal cord. Treatment of chicken embryos with bicuculline or muscimol between E5 and E8 (or between E8 and E10), inhibits the dendritic outgrowth of motoneurons when compared to vehicle-treated embryos. The inhibitory effect of bicuculline or muscimol on the dendritic morphology of motoneurons was likely due to inhibition of GABA-driven network activity since a similar effect was also observed following reduction of network activity by Kir2.1 overexpression in the spinal cord. The inhibitory effect of bicuculline or muscimol was not caused by an adverse effect on cell survival. Surprisingly, chronic treatment of chicken embryos with bicuculline or muscimol has no effect on the shape and duration of the episodes of spontaneous activity, suggesting that maturation of network activity is not altered by disruption of the dendritic outgrowth of motoneurons.

**Conclusions:**

Taken together, these findings indicate that excitatory GABA receptor activation regulates the maturation of dendritic morphology in the developing spinal cord by an activity-dependent mechanism. However, inhibition of dendritic outgrowth caused by disruption of GABA-driven activity does not alter the maturation of spontaneous electrical activity generated by spinal cord networks, suggesting that compensatory mechanisms can reverse any adverse effect of dendritic morphology on network function.

## Background

GABA (gamma-aminobutyric acid) is a major inhibitory neurotransmitter in the adult nervous system that evokes membrane hyperpolarization through activation of GABA_A _receptors. GABA-evoked hyperpolarization of the membrane potential is mediated by the opening of a Cl^- ^conductance and the subsequent influx of Cl^- ^ions. During embryonic development, however, GABA receptor activation often generates membrane depolarizations and a subsequent increase in intracellular Ca^2+ ^[1,2]. The depolarizing effect of GABA in immature neurons is caused by the presence of a high concentration of intracellular Cl^-^, which creates an outward Cl^-^gradient [3]. The bumetanide-sensitive Na^+^/K^+^/2Cl^- ^cotransporter, NKCC1, is responsible for the accumulation of Cl^- ^ions in developing chicken motoneurons [3]. Upregulation of the neuron-specific Cl^- ^transporter KCC2 is primarily responsible for lowering the intracellular Cl^- ^concentration in matured neurons [4]. Lowering the intracellular Cl^- ^concentration results in a shift in the Cl^- ^reversal potential towards more hyperpolarizing potentials and the conversion of the excitatory effect of GABA into inhibition.

Excitatory GABAergic neurotransmission is involved in the generation of spontaneous electrical activity in the spinal cord. Spontaneous electrical activity in the chicken spinal cord is produced by a network generator that relies on recurrent excitation and post-episode depression, which drives the activation of spinal motoneurons [5]. The neuronal circuits that generate spontaneous activity at early stages of spinal cord development (between embryonic day 4 (E4) and E6) rely on cholinergic and GABA neurotransmission [6]. At later stages of development (E10), however, spinal cord network activity appears to be driven by glutamate and GABA [7]. During spontaneous episodes of activity, intracellular Cl^- ^decreases significantly in the dendrites of motoneurons, indicating that changes in Cl^- ^conductance evoked by GABA receptor activation are an important component of the synaptic drive responsible for the generation of spontaneous episodes [8,9]. The ability of GABA to drive the generation of network activity is explained by the depolarizing effect of GABA receptor activation [5]. In the chicken spinal cord, the depolarizing effect of GABA receptor activation persists until E15 [10].

GABA-driven network activity promotes appropriate target projection and ion channel expression in the chicken embryo. For example, inhibition of GABAergic neurotransmission prevents motor axonal guidance [11]. Similarly, GABA-driven network activity regulates the electrical differentiation of spinal motoneurons, including Ca^2+^-dependent and A-type K^+ ^channel expression [12,13]. Little is known regarding the role of GABA-mediated activity in regulating the morphological maturation of spinal neurons. However, maturation of dendritic morphology is an important aspect of neuronal differentiation, which can ultimately regulate network function by allowing the establishment of appropriate synaptic connections with various network components. We have demonstrated that chicken lumbar motoneurons undergo considerable changes in their dendritic morphology between E6 and E11 [14]. Maturation of the dendritic morphology of motoneurons allows the establishment of sensorymotor connections and the generation of spinal cord reflexes [15,16]. The aim of this work is to investigate the role of depolarizing GABA-driven activity on the development of dendritic morphology and network function in the chicken spinal cord.

## Methods

### *In ovo *manipulations of embryonic development

Embryos were windowed at E5 or E8 and sealed with Blendoderm surgical tape (3 M Corp). Muscimol (0.1 mg/day) and bicuculline (0.3 or 0.6 mg/day) were dissolved in sterile Tyrode's buffer containing (in mM): NaCl (139), KCl (3), MgCl_2 _(1), CaCl_2 _(3), NaHCO_3 _(17). Controls consisted of embryos treated with vehicle (Tyrode's buffer). A 50-μL volume of each drug or vehicle was applied daily onto the vascularized chorioallantoic membrane as previously described by Martin-Caraballo and Dryer [12]. Considering a passive distribution of muscimol throughout the egg and an egg's volume equal to 60 mL, the concentration of muscimol used is equivalent to approximately 5 μM. The doses of bicuculline applied correspond to a final concentration of approximately 10 and 20 μM in the egg, which is sufficient to block spontaneous activity in the isolated spinal cord *in vitro*. In this study drugs were applied either between E5 and E8 or between E8 and E10. Drugs were applied daily in order to maintain a constant supply to the embryos.

### Viral infections of chicken embryos with Kir2.1

Kir2.1 inward rectifying potassium channels were expressed in the chicken spinal cord using the RCASBP(B) viral vectors, as previously described by Yoon *et al*. [2]. Briefly, pathogen-free eggs were obtained from SPAFAS (Charles River Laboratories, Wilmington, MA, USA) and incubated at 37°C. Prior to viral injections, a small window was cut in the shell directly above the embryo. Concentrated viral stocks were injected into the neural tube of E2 chicken embryos (corresponding to stage 8 to 10) using a fine tip pipette. Embryos were infected with the viral constructs RCASBP(B), RCASBP(B)-GFP or RCASBP(B)-Kir2.1. After injections, the window was closed with Scotch tape (3 M, St Paul, MN, USA) and embryos were returned to the incubator. Embryos were incubated in a humidified incubator at 37°C until E10.

### Extracellular recordings of spinal cord activity

Chicken embryos were isolated at E8 or E10 and the lumbar spinal cord was dissected in a cool (15°C) oxygenated Tyrode's solution supplemented with 12 mM glucose (see above). In order to promote tissue recovery after dissection, the spinal cord was transferred to a recording chamber and kept overnight while perfusing with cool (17°C) oxygenated Tyrode's solution. The following morning, the spinal cord was warmed for 1 hour by perfusing with Tyrode's solution at room temperature. After 1 hour, the temperature of the preparation was raised again to 27°C in order to induce the generation of spontaneous network activity. Thus, we should point out that electrical activity in the isolated preparations was elicited by warming of the perfusing solution rather than supplementing this solution with a higher extracellular K^+ ^concentration. Spinal cord activity was recorded using an extracellular electrode inserted in the motoneuron pool. Electrodes with 4 to 5 Mohm resistance were filled with a 145 mM NaCl solution (occasionally we also add DiI to the pipette solution in order to identify the location of the electrode in the spinal cord). Extracellular activity was recorded with an Axon patch amplifier after compensation of pipette junction potentials. Drugs were applied to the Tyrode's solution used to perfuse the isolated spinal cord.

### Assessment of dendritic morphology

Dendritic morphology was assessed as previously described by Ni and Martin-Caraballo [14]. Briefly, embryos were fixed in 4% paraformaldehyde in phosphate buffered saline (PBS) overnight. After fixation, a small amount of DiI (2 to 4 μL) was applied using a picospritzer (Parker, Fairfield, NJ, USA) onto the nerves in the ischiadic plexus and embryos were returned to the incubator for up to 8 weeks to allow time for complete neuronal labeling [14]. Motoneurons of the ischiadic plexus can be found throughout lumbar segments L4 to L8 [17,18]. Spinal cord tissue was sectioned into 200 μm slides and imaged with a Nikon Eclipse E600 microscope. Individually labeled motoneurons were traced using a computer-assisted camera morphometric program (Neurolucida, Microbrightfield Inc., Colchester, VT, USA).

Three criteria were used in selecting appropriate motoneurons for tracing. First, only motoneurons with their dendritic tree within a 200-μm section were included in our analysis. Motoneurons with cut dendrites on the transverse plane were not included in our analysis. We did not analyze dendrites extending in the rostro-caudal direction (see below). Second, only motoneurons sufficiently separated from their neighbors were used for tracing. We have determined that the key to successful tracing is to apply a small amount of DiI in each nerve, which will only result in the labeling of at most three motoneurons per section. Extensive application of DiI resulted in labeling of a large number of neurons, which hindered the visualization and tracing processes of single motoneurons. Third, only motoneurons with primary dendrites distributed more than 180 degrees around the cell body were considered for analysis. This was an indication that DiI has spread evenly in all directions within the cell. Neurons with primary dendrites distributed less than 180 degrees around the cell body were not considered for analysis since they were considered the result of non-uniform filling (10 to 20% of all labeled neurons). The following parameters were measured in DiI-labeled motoneurons: dendritic arbor/cell, number of primary dendrites, number of nodes (branch points), and number of ends. We should mention that our technique for assessing dendritic morphology only included dendrites located in the transverse plane of the spinal cord but obviously did not include dendrites extending in the rostro-caudal direction. Therefore, our definition of dendritic arbor/cell refers to the length of all dendritic segments lying exclusively within the transverse plane of the spinal cord section. To investigate whether changes in the dendritic tree are localized to a particular area of the arbor, we assessed dendritic length and/or number according to branch order. In this analysis, we compared the total length of primary dendrites, followed by second order dendrites (or dendrites bifurcating from primary dendrites) and so on. This allowed us to determine whether any changes in dendritic length may occur in proximal dendrites or more distal dendrites. Changes in soma morphology were assessed by measuring cell body perimeter and somatic surface area. The cell body perimeter was measured by focusing on the plane of the cell body, where cell dimensions were the greatest, and outlining the cell body contour. The values for the somatic surface area were automatically generated by the morphometric software (Neurolucida) from the readings of the cell body contour.

### Islet1/2 immunohistochemistry and design-based stereology

Motoneuron survival was quantified by assessing the number of Islet1/2-positive cells in the first six segments of the lumbar enlargement as previously described by Yoon *et al*. [2]. Briefly, lumbar segments L1 to L6 were removed at E10 and fixed in Zamboni's fixative (4% paraformaldehyde plus 15% picric acid in 0.1 M PBS) at 4°C overnight, washed three times in PBS, and equilibrated in 30% sucrose/PBS overnight. Spinal cord tissue was embedded in OCT freezing medium, and 30-μm cryostat sections were serially collected using a Leica cryostat. After air drying and post fixation, slides were washed three times in 0.1 M PBS and blocked overnight in blocking solution (PBS containing 10% horse serum and 0.5% Triton X-100) at 4°C. Sections were incubated overnight at 4°C with a mouse anti-Islet1 (1:100 hybridoma supernatant, clone 39.4D5, Developmental Studies Hybridoma Bank, University of Iowa) diluted in blocking solution. This antibody recognizes both Islet1 and 2 [19,20]. Following three washes with PBS, sections were incubated with 0.5% hydrogen peroxide for 30 minutes to block endogenous peroxidase activity. After three more washes with PBS, slides were incubated for 2 hours at room temperature with a biotinylated goat anti-mouse antibody (1:500, Vector Laboratories, Burlingame, CA, USA). Following three washes with PBS, slides were incubated with Vectastain ABC-HRP solution for 3 to 4 hours at room temperature. Islet staining was visualized by using a nickel/cobalt enhanced diaminobenzidine solution. After three washes, slides were mounted using AquaMount (Lerner Laboratories, Pittsburgh, PA, USA). The number of Islet-positive neurons on both sides of the ventral spinal cord was counted in every fifth section using StereoInvestigator software (Microbrightfield Inc., Williston, VT, USA). Images were obtained with a Nikon Eclipse E600W microscope coupled to a MicroFire video camera (Optronics) and with an x, y, z stage drive and position transducer (MAC 2000, Ludl Electronic Products, Hawthorne, NY, USA). Under low magnification, the boundary of the motoneuron pool was identified and the boundary contour was drawn using the software-pointing device. A randomly generated sampling grid was placed over the contour area, containing 5 to 10 square counting frames (175 × 175 μm). Only Islet-stained nuclei within the counting frame and not in contact with exclusion lines were counted using a 40× objective. The total number of motoneurons was obtained by adding together all counted neurons between L1 and L6 spinal segments and multiplying by five.

### Data analysis

Values are presented as mean ± standard error of the mean where indicated. Statistical analyses consisted of one-way ANOVA followed by *post hoc *analysis using Tukey's honest significant difference test for unequal *n *for comparisons between multiple groups (SigmaStat Software, San Jose, CA, USA). Throughout, *P *≤ 0.05 was regarded as significant.

## Results

**Figure 1:**
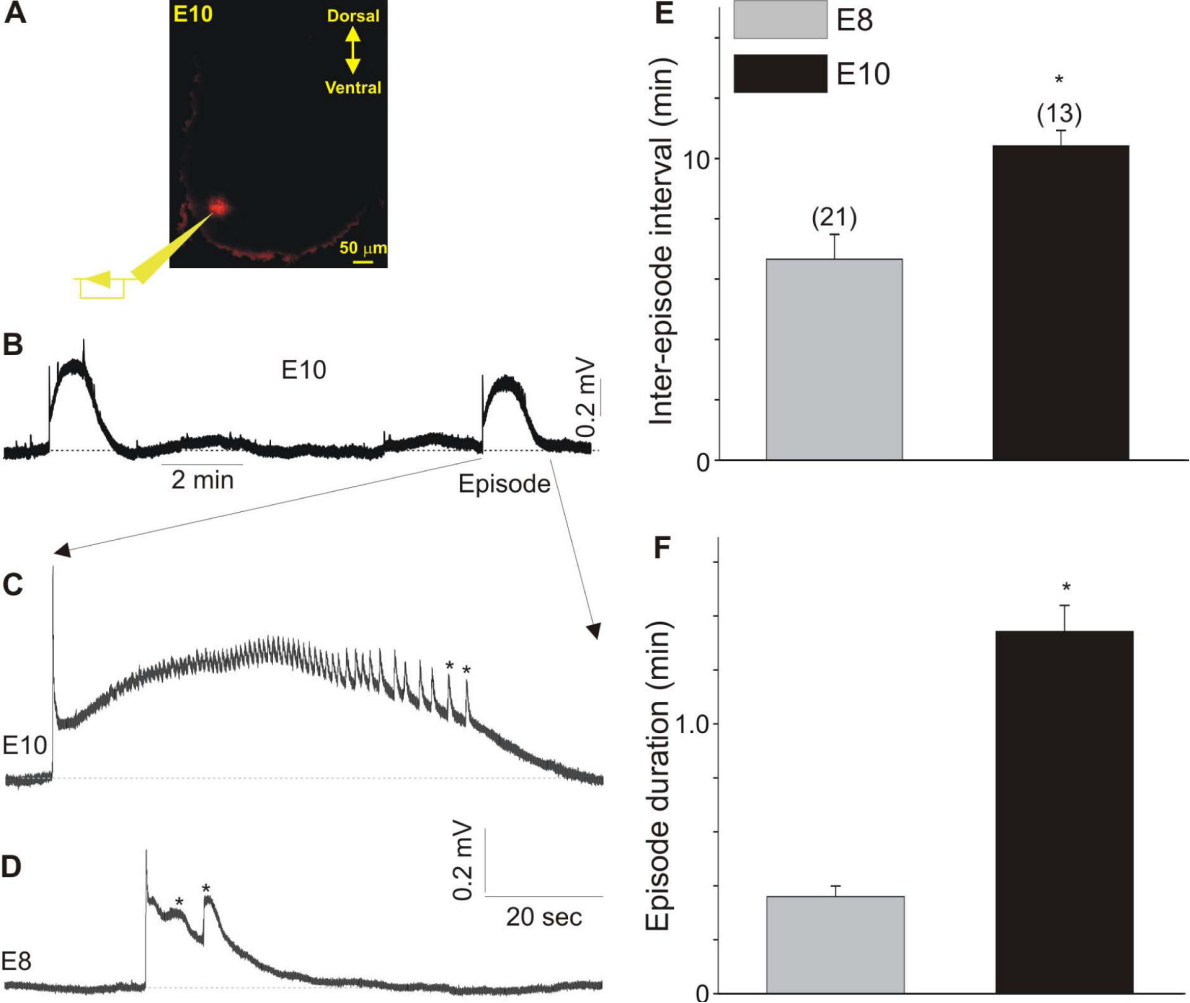
**Network activity in the E8 and E10 chicken spinal cord**. **(A) **Fluorescent image of a transverse spinal cord section showing the location of the recording electrode in the motoneuron pool as indicated by the location of the DiI fluorescent spot. **(B) **Typical trace obtained by recording spontaneous activity in an E10 spinal cord. **(C) **Expanded trace of spontaneous activity generated in an E10 chicken spinal cord. Each episode of activity consisted of a fast rising component that develops into a series of bursts (asterisks). **(D) **Spontaneous activity generated in an E8 chicken spinal cord. The bursts of activity within each episode at E8 (asterisks) are small and long lasting when compared with bursts generated at E10. Notice that the episode duration was shorter than that generated in E10 spinal cords and consisted of a few bursts (asterisk). **(E, F) **Comparison of episode duration (E) and inter-episode interval (F) in E8 and E10 chicken spinal cords. In (E, F), asterisks represent *P *< 0.05 versus E8.

The period spanning between E6 and E11 is critical for the maturation of network activity in the chicken spinal cord [21]. In order to assess the effect of disrupting GABAergic neurotransmission on network activity and dendritic outgrowth, we first characterized the pattern of spontaneous activity in the chicken spinal cord at E8 and E10. Changes in the pattern of spontaneous activity in the spinal cord were studied by extracellular recordings of electrical signals generated in the motoneuron pool of isolated spinal cords from E8 and E10 chicken embryos (Figure 1). Recordings were performed by inserting an electrode into the motoneuron pool and recording the extracellular signals generated by bursts of spontaneous activity in the lumbosacral segments 4 and 5. To confirm the location of the recording electrode within the motoneuron pool, we applied a small amount of DiI to the pipette solution (Figure 1A). As represented in Figure 1A, DiI was found in a small portion of the ventral spinal cord where the lumbar motoneurons are located. Recording of spontaneous activity in E10 chicken spinal cords indicates that the pattern of activity is similar to that previously recorded with suction electrodes from spinal cord nerves (Figure 1B, C) [7,22]. At E10, episodes of spontaneous activity were generated once every 10 minutes on average and lasted for ≤ 1.4 minutes (Figure 1B, C). Each episode generated at E10 consisted of an initial fast rising component followed by multiple bursts of activity (asterisks in Figure 1C). These bursts of activity within an episode are likely generated by motoneuron firing [7]. At E8, each episode consisted of an initial fast rising component followed by one to two long-lasting bursts (asterisks in Figure 1D). Episodes in E8 spinal cords were generated at a faster rate when compared with the episodes at E10 (inter-episode interval at E8 approximately 6 minutes; Figure 1E). Episode duration in E8 spinal cords was also significantly shorter when compared with the episode duration at E10 (≤ 0.4 minutes; Figure 1F).

**Figure 2:**
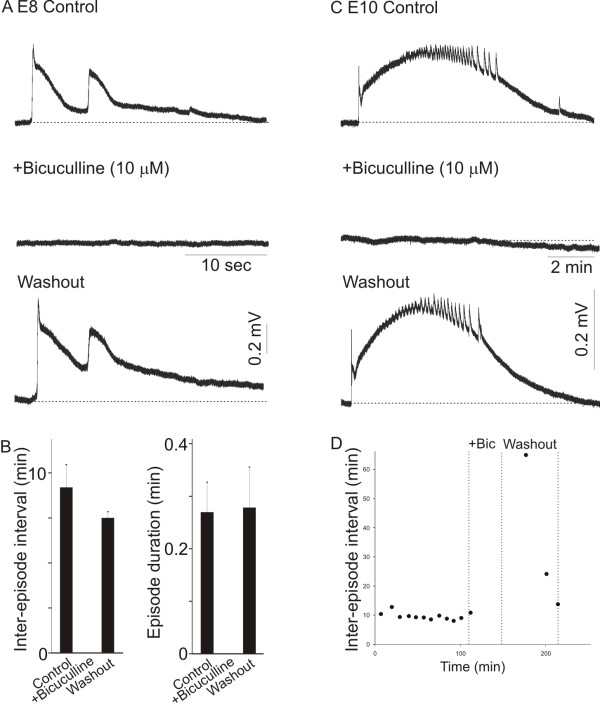
**Role of GABAergic neurotransmission on the generation of network activity in the E8 and E10 chicken spinal cord**. **(A, B) **Effect of bicuculline on the generation of network activity in the E8 chicken spinal cord. Network activity in the E8 chicken spinal cord was completely eliminated by treatment with 10 μM of bicuculline. Notice that activity recovers following washout of bicuculline. (B) Data showing the effect of bicuculline on the inter-episode interval and episode duration in E8 chicken spinal cords (n = 4). After washout of bicuculline from the bathing solution, the inter-episode interval and episode duration recovers to control levels. **(C, D) **Network activity in the E10 chicken spinal cord was completely eliminated by treatment with 10 μM of bicuculline. After washout of bicuculline, activity recovers to control levels. (D) Time-dependent effect of bicuculline (Bic) on the inter-episode interval in an E10 spinal cord. Notice that application of bicuculline for 30 minutes causes a complete cessation of network activity, which tends to recover to control levels after removal of bicuculline from the bathing solution.

Excitatory GABAergic neurotransmission plays a critical role in driving the generation of spontaneous activity in the spinal cord [5,6]. To assess whether the concentrations of bicuculline and muscimol used to disrupt dendritic outgrowth were optimal in disrupting the generation of spontaneous activity, we treated the isolated spinal cords of E8 or E10 chicken embryos with 10 μM bicuculline or 5 μM muscimol. Exposure of spinal cords to 10 μM bicuculline (for 30 minutes to 3 hours) was sufficient to evoke a complete inhibition of network activity in both E8 (Figure 2A, B) and E10 spinal cords (Figure 2C, D). Both at E8 (Figure 2A) and E10 (Figure 2C), washout of bicuculline from the bathing solution allowed activity to recover to control levels. In all ages tested, measurements of the inter-episode interval and episode duration reveal that bicuculline caused a complete elimination of network activity that recovers to normal levels after washout of the drug from the bathing solution (Figure 2B, D). As previously shown [12,23], muscimol (5 μM) also caused a complete inhibition of spontaneous activity in the isolated spinal cord preparation at E8 and E10 (not shown).

**Figure 3:**
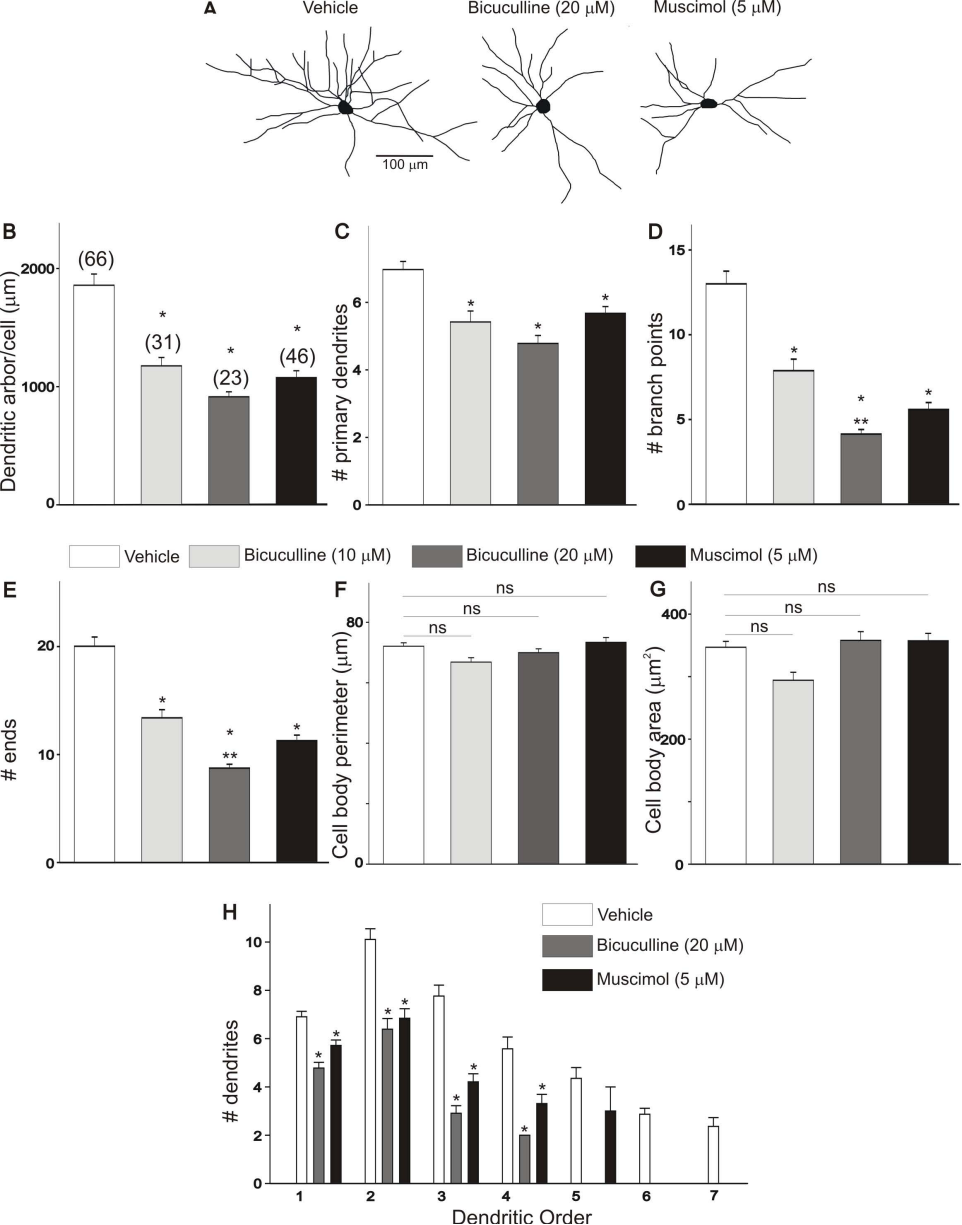
**Effect of bicuculline and muscimol on the dendritic morphology of E8 chicken lumbar motoneurons**. The effect of bicuculline and muscimol on the dendritic morphology of E8 chicken spinal motoneurons was tested following daily applications of these drugs between E5 and E8. **(A) **Neurolucida drawing of DiI-labeled E8 lumbar motoneurons showing the typical dendritic structure of vehicle-, bicuculline- and muscimol-treated motoneurons. **(B-E) **Treatment of chicken embryos with bicuculline (10 or 20 μM) or muscimol (5 μM) between E5 and E8 causes a significant decrease in the dendritic complexity of E8 motoneurons. Notice significant reduction in the overall length of the dendritic arbor/cell, number of primary dendrites, number of branch points and number of ends. **(F, G) **Treatment of chicken embryos with bicuculline (10 or 20 μM) or muscimol (5 μM) between E5 and E8 has no effect on the cell body morphology. **(H) **Comparison of E8 dendritic morphology as a function of dendritic order in embryos treated with bicuculline or muscimol between E5 and E8. Application of bicuculline or muscimol to chicken embryos between E5 and E8 causes a significant reduction of the number of dendrites between the first and fourth dendritic orders and a complete elimination of higher order dendrites (sixth and seventh dendritic orders) when compared to vehicle-treated embryos. *P < 0.01 versus vehicle; **P < 0.05 versus bicuculline 10 μM. NS denotes no significant differences between the groups as indicated by one-way ANOVA.

The period spanning between E6 and E11 is important for the maturation of the dendritic morphology of spinal motoneurons. Our previous results indicate that between E6 and E11 there is a significant increase in the dendritic length and complexity of chicken lumbar motoneurons [14]. Thus the question arises: what is the effect of excitatory GABAergic neurotransmission on the maturation of dendritic morphology of lumbar motoneurons? To characterize changes in the morphology of lumbar motoneurons following disruption of GABAergic neurotransmission, we traced individual DiI-labeled motoneurons. In order to determine whether disruption of GABAergic neurotransmission alters the dendritic morphology of E8 motoneurons, we applied daily doses of bicuculline (10 or 20 μM/day) or muscimol (5 μM/day) to the chorioallantoic membrane of chicken embryos. Controls consisted of chicken embryos treated with vehicle. Drugs were applied between E5 and E8 and embryos were isolated 6 hours after the last drug application on E8. Dendritic morphology was studied following injection of a small amount of DiI into the ischiadic plexus, which innervates the muscles of the hindlimb and lower leg in the chicken [17]. Examples of DiI-traced motoneurons from E8 chicken embryos treated with vehicle, bicuculline or muscimol are represented in Figure 3A. Treatment of chicken embryos with bicuculline (10 or 20 μM/day) or muscimol (5 μM/day) between E5 and E8 caused a significant decrease in the length of the dendritic arbor/cell of E8 motoneurons when compared with vehicle-treated embryos (Figure 3B). The reduction in the length of the dendritic arbor/cell was the result of a decrease in the dendritic complexity of E8 motoneurons as indicated by the reduction in the number of primary dendrites (Figure 3C), the number of branch points (Figure 3D) and the reduced number of ends (Figure 3E). Disruption of GABAergic neurotransmission did not have any effect on cell body perimeter and somatic surface area (Figure 3F, G). These data suggest that disruption of GABAergic neurotransmission between E5 and E8 has important implications for the maturation of the dendritic morphology of motoneurons. Disruption of GABA-mediated activity in the spinal cord causes a significant delay in the normal maturation of dendritic morphology in developing motoneurons.

To determine whether changes in the dendritic morphology of E8 motoneurons occur in specific segments of the dendritic tree, we analyzed changes in the number of dendrites as a function of dendritic order. This analysis is useful in assessing whether changes in dendritic morphology occur in proximal or distal dendrites. The total number of dendrites within each segment was plotted as a function of their branch order (Figure 3H). As represented in Figure 3H, there is a significant decrease in the number of dendrites located between the first and fourth dendritic orders in bicuculline- (20 μM) and muscimol-treated (5 μM) motoneurons when compared with vehicle-treated motoneurons. We also observed that the E8 motoneurons of vehicle-treated embryos had higher order dendrites than those found in bicuculline- or muscimol-treated embryos. For example, more dendritic segments were found in vehicle-treated embryos than in bicuculline- or muscimol-treated embryos at dendritic orders between the fifth and seventh (Figure 3H). These data suggest that the decrease in the dendritic morphology of E8 motoneurons following disruption of GABAergic neurotransmission is the result of a significant rearrangement of dendritic branching within each dendritic order.

**Figure 4:**
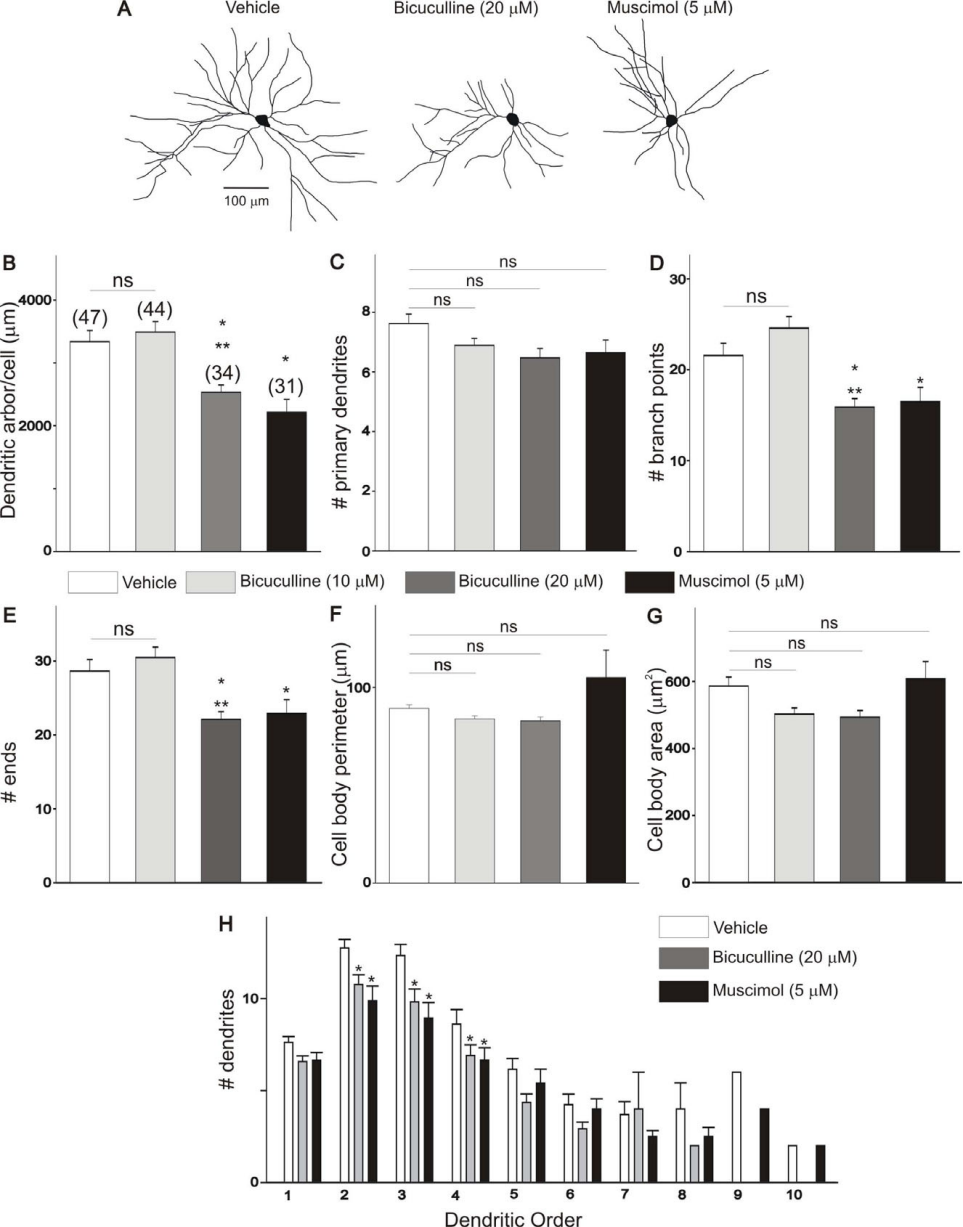
**Effect of bicuculline and muscimol on the dendritic morphology of E10 chicken lumbar motoneurons**. Changes in the dendritic morphology of E10 chicken spinal motoneurons were assessed following daily applications of bicuculline and muscimol between E8 and E10. **(A) **Neurolucida drawing of DiI-labeled E10 lumbar motoneurons showing the typical dendritic structure of vehicle- and drug-treated motoneurons. **(B-E) **Treatment of chicken embryos with bicuculline (20 μM) or muscimol (5 μM) between E8 and E10 causes a significant decrease in the dendritic complexity of E10 motoneurons. Notice significant reduction in the overall length of the dendritic arbor/cell, number of branch points and number of ends in bicuculline- or muscimol-treated embryos when compared with vehicle-treated embryos. Treatment of chicken embryos with bicuculline (10 μM) has no effect on the dendritic morphology of E10 motoneurons when compared with vehicle-treated embryos. **(F, G) **Treatment of chicken embryos with bicuculline (10 or 20 μM) or muscimol (5 μM) between E8 and E10 has no effect on the cell body morphology. **(H) **Comparison of E10 dendritic morphology as a function of dendritic order in embryos treated with bicuculline or muscimol between E8 and E10. Application of bicuculline or muscimol to chicken embryos between E8 and E10 causes a significant reduction in the number of dendrites between the second and fourth dendritic orders when compared to vehicle-treated embryos. *P < 0.01 versus vehicle; **P < 0.05 versus bicuculline 10 μM. NS denotes no significant differences between the groups as indicated by one-way ANOVA.

Similar results were also obtained when the dendritic morphology of lumbar motoneurons was assessed at E10 following bicuculline or muscimol treatment of chicken embryos between E8 and E10. To investigate how disruption of GABA-mediated activity alters the maturation of the dendritic morphology of E10 motoneurons, chicken embryos were treated *in ovo *with daily doses of bicuculline (10 to 20 μM/day) or muscimol (5 μM/day) between E8 and E10. As represented in Figure 4A-H, disruption of GABAergic neurotransmission with bicuculline or muscimol between E8 and E10 resulted in a significant change in the dendritic morphology of E10 motoneurons. Typical Neurolucida drawings of E10 motoneurons from control- and drug-treated embryos are represented in Figure 4A. Treatment of chicken embryos with either bicuculline (20 μM/day) or muscimol (5 μM/day) between E8 and E10 caused a significant decrease in the length of the dendritic arbor/cell when compared with vehicle-treated embryos (Figure 4B). In contrast to the effect observed in E8 motoneurons, disruption of GABA neurotransmission with bicuculline or muscimol has no effect on the number of primary dendrites of E10 motoneurons when compared with vehicle-treated embryos (Figure 4C). The reduction in the length of the dendritic arbor/cell was the result of a decrease in the dendritic complexity of motoneurons as indicated by the reduction in the number of branch points (Figure 4D) and the reduced number of dendritic ends (Figure 4E). Interestingly, treatment of chicken embryos with a lower concentration of bicuculline (10 μM/day) between E8 and E10 has no effect on the dendritic morphology of E10 motoneurons (Figure 4B-E). Thus, the effect of 10 μM/day bicuculline on the number of primary dendrites in E10 chicken embryos differs from that seen in E8 chicken embryos (Figure 3B-E). These differences could be mediated by changes in drug accessibility in the embryos and/or age-dependent changes in the GABA receptor properties (see Discussion). Disruption of GABAergic neurotransmission has no effect on the cell body morphology of E10 motoneurons (including cell body perimeter and somatic surface area; Figure 4F-G). These data suggest that disruption of GABAergic neurotransmission during a specific period of development has important implications for the maturation of the dendritic morphology of motoneurons.

To determine whether changes in the dendritic morphology of E10 motoneurons occur in specific segments of the dendritic tree, we analyzed changes in the number of dendrites as a function of dendritic order. The total number of dendrites within a segment was plotted as a function of their branch order (Figure 4H). As represented in Figure 4H, there is a significant decrease in the number of dendrites located between the second and fourth dendritic orders in bicuculline- (20 μM) and muscimol-treated (5 μM) embryos when compared with vehicle. We also observed that the E10 motoneurons of vehicle-treated embryos had higher order dendrites than those found in bicuculline-treated embryos. For example, more dendritic segments were found in vehicle-treated embryos than in bicuculline-treated embryos. These data suggest that the decrease in the dendritic morphology of E10 motoneurons following disruption of GABAergic neurotransmission is the result of a significant rearrangement of dendritic branching within each dendritic order.

Since bicuculline and muscimol inhibit the generation of spontaneous activity in the chicken spinal cord, it is possible that the inhibitory effect on dendritic outgrowth is caused by disruption of the normal pattern of network activity in the developing chicken embryo. In order to investigate this possibility, we infected chicken embryos with an RCABP(B) viral vector carrying the inward rectifier K^+ ^channel Kir2.1. Controls consisted of non-infected chicken embryos or embryos infected with an RCABP(B)-GFP construct. As we have previously reported, the avian replication-competent retroviral vector RCASBP(B) allows the stable expression of functional Kir2.1 channels in spinal neurons [2]. Kir2.1 channel expression decreases the resting membrane potential and the input resistance of the membrane, which dampen electrical excitability in Kir2.1-expressing motoneurons [2]. This leads to a significant reduction in spontaneous motor activity in developing chicken embryos [2]. In agreement with our previous findings [2], infection of chicken embryos with the RCABP(B)-Kir2.1 construct resulted in a significant reduction in the number of spontaneous kicks generated during a 3-minute interval in chicken embryos isolated at E8 or E10 when compared with non-infected embryos (E8 control = 12.6 ± 0.4, n = 22; E8 RCABP(B)-Kir2.1 = 1.1 ± 0.3, n = 18, *P *< 0.05 versus E8 control; E10 control = 25.3 ± 1.2, n = 13; E10 RCABP(B)-Kir2.1 = 5.1 ± 0.6, n = 14, *P *< 0.05 versus E10 control).

**Figure 5:**
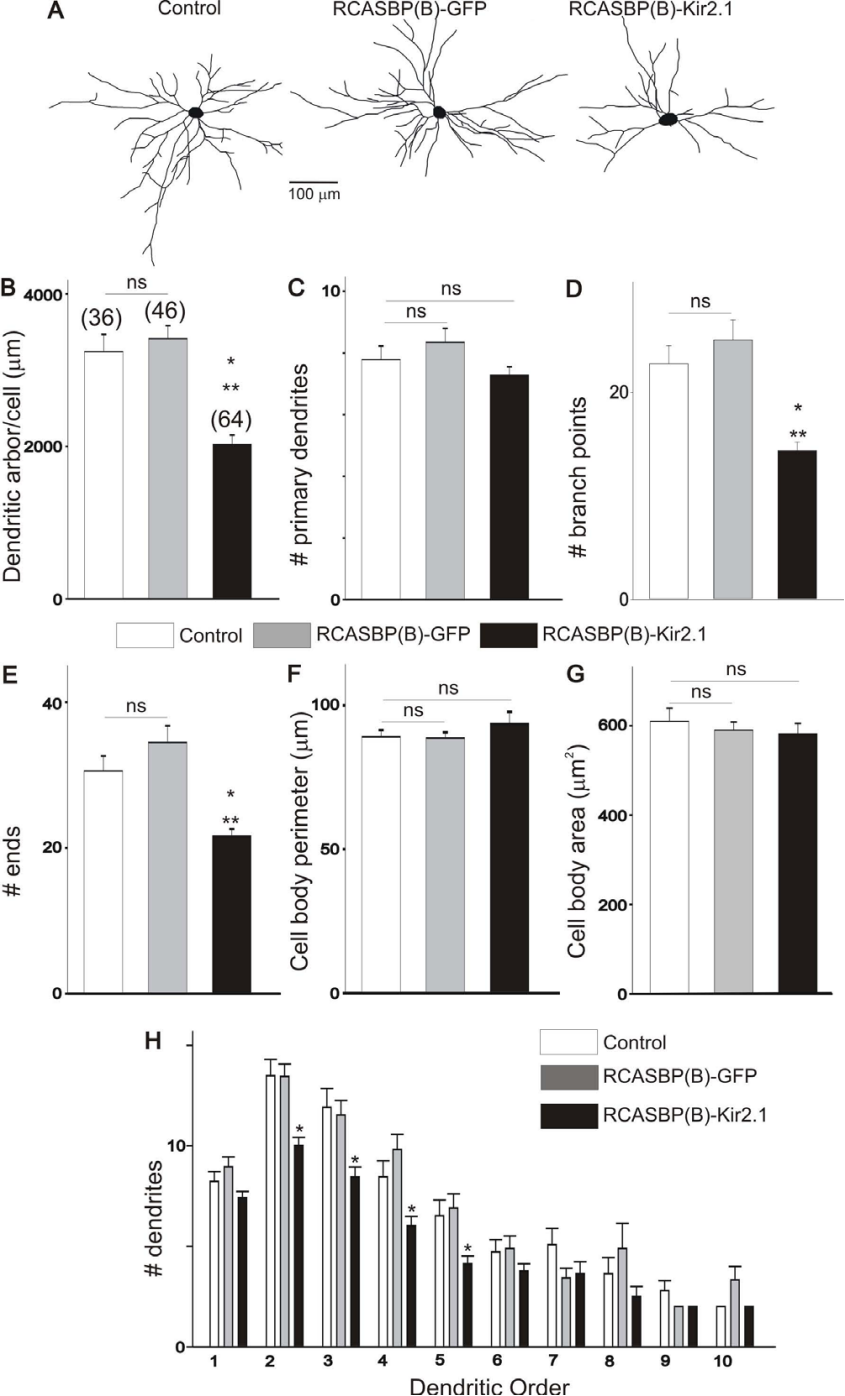
**Disruption of electrical excitability with the Kir2.1 inward rectifying K^+ ^channel causes a significant reduction in dendritic morphology of E10 lumbar motoneurons**. To study how disruption of electrical excitability alters the maturation of the dendritic morphology of the motoneurons, chicken embryos were infected with the RCASBP(B)-GFP or RCASBP(B)-Kir2.1 viral constructs at E2. Embryos were isolated at E10 and prepared for tracing of the dendritic tree of the motoneurons. **(A) **Typical Neurolucida drawing of motoneurons, traced from control (non-infected) or RCASBP(B)-GFP or RCASBP(B)-Kir2.1 infected chicken embryos. **(B-E) **Inhibition of electrical excitability in RCASBP(B)-Kir2.1 infected embryos causes a significant reduction in the dendritic complexity of E10 motoneurons as indicated by the changes in the overall length of the dendritic arbor/cell, the number of branch points and the numbers of ends when compared with control or RCASBP(B)-GFP infected embryos. **(F, G) **Infection of chicken embryos with RCASBP(B)-GFP or RCASBP(B)-Kir2.1 has no effect on the cell body morphology of E10 motoneurons. **(H) **Comparison of E10 dendritic morphology as a function of dendritic order in embryos infected with RCASBP(B)-GFP or RCASBP(B)-Kir2. Infection of chicken embryos with RCASBP(B)-Kir2 causes a significant reduction in the number of dendrites between the second and fifth dendritic orders when compared to control or RCASBP(B)-GFP infected embryos. *P < 0.01 versus control (non-infected embryos); **P < 0.05 versus RCASBP(B)-GFP infected embryos. NS denotes no significant differences between the groups as indicated by one-way ANOVA.

Infection of chicken embryos with the RCABP(B)-GFP construct has no effect on the dendritic morphology of E10 motoneurons when compared with age-matched motoneurons from non-infected embryos (Figure 5A-D). However, tracing of DiI-labeled motoneurons from chicken embryos infected with the RCABP(B)-Kir2.1 construct show a significant reduction in the dendritic complexity when compared with control embryos (non-infected embryos or embryos infected with RCABP(B)-GFP). Thus, infection of chicken embryos with RCABP(B)-Kir2.1 evoked a 46% reduction in the length of the dendritic arbor/cell when compared with RCABP(B)-GFP-infected embryos (Figure 5B). These changes in dendritic length are likely mediated by a significant decrease in the number of branch points (Figure 5D) and the number of terminal dendrites (Figure 5E). Infection of chicken embryos with RCABP(B)-Kir2.1 and the reduction of spontaneous activity has no effect on the number of primary dendrites (Figure 5C) or cell body morphology (Figure 5F, G). In order to investigate which dendritic branches are most likely affected by infection of chicken embryos with the RCABP(B)-Kir2.1 construct, we plotted the number of dendrites as a function of branch order (Figure 5H). Our results show that infection of chicken embryos with the RCABP(B)-Kir2.1 construct causes a significant decrease in the number of dendrites between the second and fifth dendritic orders when compared with control or RCABP(B)-infected embryos. These data suggest that disruption of the electrical excitability of motoneurons during a specific period of development alters the morphological maturation of the dendritic tree.

**Figure 6:**
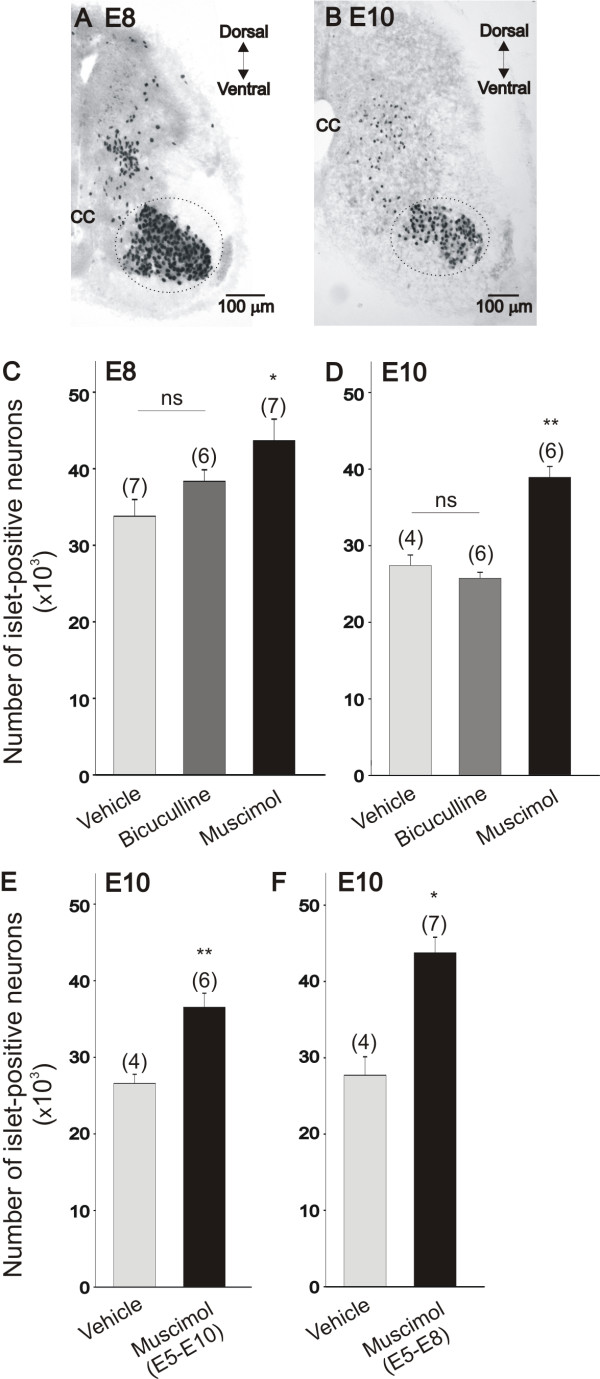
**Muscimol and bicuculline have no toxic effect on motoneuron survival in the chicken spinal cord**. **(A, B) **Islet1/2-staining in the lumbar spinal cord of E8 and E10 chicken embryos. Only Islet1/2-positive neurons in the motoneuron pool were counted (circled area). Islet1/2-positive interneurons located in dorsal and medial portions of the spinal cord were not included in our measurements. CC = central canal. **(C, D) **Number of Islet-positive neurons in the lumbar spinal cord of E8 (C) and E10 (D) chicken embryos in control (vehicle) and following treatment *in ovo *with bicuculline or muscimol. Bicuculline did not affect the number of Islet-positive neurons at E8 or E10, whereas muscimol causes a significant increase in the number of surviving motoneurons. The number of Islet-positive neurons in the E8 lumbar spinal cord was assessed following *in ovo *treatments with bicuculline (20 μM/day) or muscimol (5 μM/day) between E5 and E8. The number of Islet-positive neurons at E10 was assessed following *in ovo *drug treatments between E8 and E10. **(E, F) **The stimulatory effect of muscimol on motoneuron survival was sustained. Drug-treatment from E5 to E10 (E) or from E5 to E8 (F) resulted in similar number of Islet-positive neurons at E10. *P < 0.05 versus E8 vehicle, **P < 0.05 versus E10 vehicle. NS denotes no significant differences between the groups as indicated by one-way ANOVA.

In order to determine whether the inhibitory effect of bicuculline and muscimol on dendritic outgrowth was caused by a negative effect of the drugs on motoneuron survival, we counted the number of Islet1/2-positive neurons in the ventral spinal cord using design-based stereology (Figure 6A, B). The LIM homeodomain transcription factor Islet1/2 is a marker of postmitotic spinal motoneurons [19,20]. We have previously demonstrated that counting the number of Islet-positive neurons in the lumbar spinal cord is a reliable technique to measure changes in cell survival [2]. Only Islet1/2-positive cells in the motoneuron pool were counted (circled area in Figure 6A, B). Islet1/2-positive interneurons located in the dorsal and medial areas of the spinal cord were not included. *In ovo *treatment of chicken embryos with bicuculline (20 μM) between E5 and E8 has no significant effect on motoneuron survival at E8 (Figure 6C). A similar effect was also obtained when embryos were treated with bicuculline between E8 and E10 (Figure 6D). In this case, the number of Islet-positive neurons in the E10 lumbar spinal cord was not significantly different between vehicle- and bicuculline-treated embryos. These findings indicate that the inhibitory effect of bicuculline on dendritic outgrowth was not caused by any adverse effect on motoneuron viability. In contrast to bicuculline, treatment of chicken embryos with muscimol (5 μM) resulted in a significant increase in the number of Islet1/2-positive neurons in the spinal cord. Thus, treatment of chicken embryos with muscimol between E5 and E8 caused a 20% increase in the number of Islet1/2-positive neurons in the E8 spinal cord (Figure 6C). Similarly, when embryos were treated with muscimol between E8 and E10, there was a 20% increase in the number of Islet1/2-positive neurons in the E10 spinal cord (Figure 6D). The effect of muscimol in promoting motoneuron survival was long-lasting and independent of the duration of drug application. Thus, we observed a comparable increase in the number of Islet1/2-positive neurons in the E10 spinal cord when chicken embryos were treated with muscimol between E5 to E10 (Figure 6E) or between E5 and E8 (Figure 6F).

**Figure 7:**
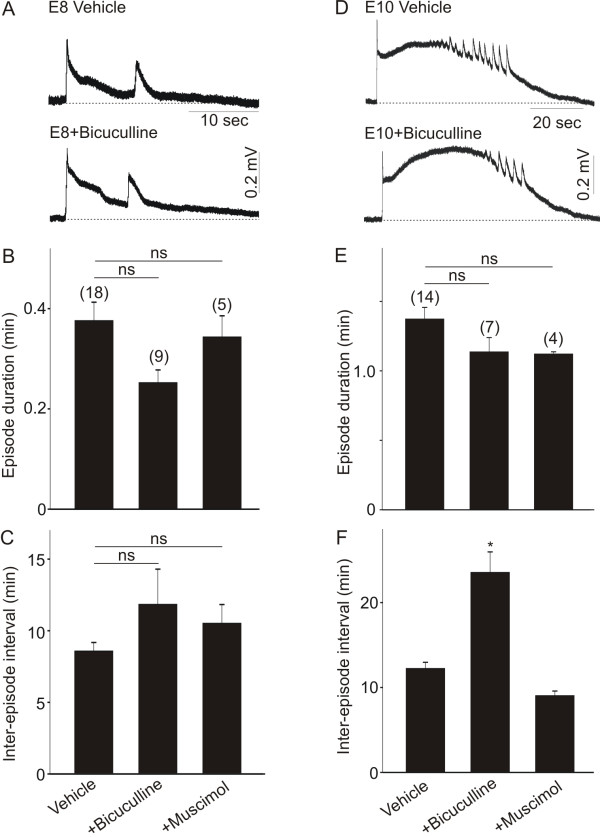
**Effect of chronic inhibition of GABAergic neurotransmission on the maturation of spontaneous network activity in the chicken spinal cord**. GABAergic neurotransmission was disrupted *in ovo *by daily applications of bicuculline (20 μM/day) or muscimol (5 μM/day). Drugs were applied to the chorioallantoic membrane of chicken embryos between E5 and E8 **(A-C) **or between E8 and E10 **(D-F)**. After the last drug application, embryos were removed (E8 or E10) and the spinal cord was isolated for recording of spontaneous network activity. (A) Typical example of an episode of spontaneous activity generated in a vehicle- and a bicuculline-treated spinal cord at E8. Notice that the overall shape of each episode remains the same after chronic inhibition of GABA receptor activation. (B, C) Chronic inhibition of GABAergic neurotransmission did not alter the episode duration and inter-episode interval in E8 spinal cords. (D) Overall shape of an episode of spontaneous activity generated in a vehicle- and a bicuculline-treated spinal cord at E10. Notice that the overall shape of each episode remains the same after chronic inhibition of GABA receptor activation. (E, F) Chronic inhibition of GABAergic neurotransmission did not alter the episode duration but causes a significant increase in the inter-episode interval at E10 (*P < 0.05 versus vehicle). NS denotes no significant differences between the groups as indicated by one-way ANOVA.

Does the altered dendritic morphology caused by disruption of GABA-driven network activity have any effect on the maturation of spontaneous activity in the chicken spinal cord? To investigate whether inhibition of dendritic morphology caused by disruption of GABA-driven activity has any effect on the maturation of network function, chicken embryos were treated with daily doses of bicuculline (20 μM/day) or muscimol (5 μM/day), whereas controls received an equal volume of vehicle solution. Embryos were treated with daily doses of bicuculline or muscimol between E5 and E8 (or between E8 and E10). After *in ovo *drug treatments, the spontaneous activity generated in the isolated spinal cord from E8 or E10 embryos was recorded. Treatment of chicken embryos with daily doses of bicuculline between E5 and E8 had no obvious effect on the overall pattern of each episode of spontaneous activity in the E8 isolated spinal cord (Figure 7A). Similarly, treatment of chicken embryos with daily doses of muscimol between E5 and E8 had no noticeable effect on the overall pattern of each episode of network activity in the E8 isolated spinal cord (not shown). Quantification of the effect of bicuculline and muscimol on the maturation of network activity in E8 spinal cords indicates that treatment of chicken embryos with daily doses of these drugs between E5 and E8 did not disrupt the episode duration (Figure 7B) or inter-episode interval (Figure 7C) when compared with vehicle-treated embryos. Similar findings were also observed in chicken embryos chronically treated with bicuculline (20 μM/day) or muscimol (5 μM/day) between E8 and E10 (Figure 7D-F). As represented in Figure 7D, treatment of chicken embryos with daily doses of bicuculline between E8 and E10 had no obvious effect on the overall shape of each episode of activity. Similarly, no effect on the overall pattern of each episode of activity was detected in the network activity generated in E10 isolated spinal cords following daily treatment of chicken embryos with muscimol between E8 and E10 (not shown). Quantification of the effect of bicuculline and muscimol on the maturation of the network activity in E10 spinal cords indicates that daily treatment of chicken embryos with bicuculline or muscimol between E8 and E10 has no effect on the episode duration (7E) when compared with vehicle-treated embryos. However, chronic exposure to bicuculline from E8 to E10 causes a significant increase in the duration of the inter-episode interval. Thus, our data demonstrate that disruption of the normal pattern of dendritic development by chronic treatment of chicken embryos with muscimol had no effect on the maturation of network activity in the chicken spinal cord. Chronic treatment of chicken embryos with bicuculline at early stages of development (E6 to E8) also had no effect on the maturation of network activity in the chicken spinal cord but altered the inter-episode interval at a later developmental stage (E8 to E10).

## Discussion

In this study we have examined the effect of GABA-driven activity on the maturation of dendritic morphology and network function during a critical period of spinal cord development. Because of the easy accessibility of the chicken embryo to manipulations *in ovo*, we were able to disrupt the normal pattern of GABA receptor activation with various pharmacological agents. The essential findings of this study are the following. First, excitatory GABA receptor activation regulates the maturation of dendritic morphology in the developing spinal cord. Second, GABAergic synaptic transmission regulates the morphological maturation of motoneurons by driving the generation of network activity in the chicken spinal cord. Third, inhibition of dendritic outgrowth caused by disruption of GABA-driven activity does not alter the maturation of spontaneous electrical activity generated by spinal cord networks, suggesting that compensatory mechanisms can reverse any adverse effect of dendritic morphology on network function.

The period spanning from E6 to E11 is critical for the functional and morphological development of the chicken neuromuscular system. At E6, lumbar motoneurons begin to innervate hindlimb muscles, which ultimately leads to the formation of functional synapses and the generation of spontaneous motor activity [6,7,24]. During this period, active spinal cord networks generate bursts of spontaneous activity that drive the maturation of lumbar motoneurons. For example, spontaneous electrical activity in the chicken lumbar spinal cord drives the electrical differentiation of spinal motoneurons by regulating the functional expression of A-type and Ca^2+^-dependent K^+ ^conductances [12,13]. Between E6 and E11, there are also considerable changes in the dendritic morphology of motoneurons [14,15], which leads to the development of sensorimotor synaptic connections between motoneurons and their sensory afferents.

Our present results demonstrate that excitatory GABA-driven activity regulates the maturation of dendritic morphology in developing motoneurons. Thus, disruption of GABA receptor activation *in ovo *alters the normal pattern of dendritic outgrowth in developing motoneurons. Treatment of chicken embryos with bicuculline or muscimol between E5 and E8 (or between E8 and E10) causes a significant reduction in dendritic outgrowth and complexity as demonstrated by changes in the number of branch points, the number of ends and the distribution of dendrites at different dendritic orders. These changes in dendritic morphology, obtained following disruption of GABA receptor activation between E5 and E8 or between E8 and E10, resulted in an overall reduction in the length and complexity of the dendritic tree. Although significant differences exist in the organization of spinal networks and the functional maturation of motoneurons between these two stages of development [6,7,12,25], our results indicate that GABAergic synaptic transmission regulates the morphological maturation of spinal motoneurons independently of the level of network maturity. Thus, we observed that both bicuculline and muscimol were effective in disrupting the dendritic morphology of motoneurons when applied either between E5 and E8 or between E8 and E10. We should point out that GABA-driven activity only targets the maturation of the dendritic morphology of the motoneurons without causing any significant change in cell body morphology. Our results also demonstrate that treatment of chicken embryos with bicuculline (10 μM/day) between E5 and E8 was more effective in causing a significant reduction in the dendritic outgrowth of motoneurons when compared with bicuculline-treated embryos between E8 and E10. These differences could arise from changes in drug accessibility at different stages of development. Alternatively, it may be an indication of developmental changes in the affinity of bicuculline to bind to GABA_A _receptors [26]. Overall, the inhibitory effect of bicuculline and muscimol on the maturation of the dendritic morphology of motoneurons demonstrates another important aspect of GABA-driven activity in regulating the development of the spinal cord *in ovo*, which could have important implications for the maturation of the neuromuscular system [11-13]. These results are also consistent with recent evidence demonstrating that disruption of excitatory GABA activity in the nervous system alters the morphological maturation of cortical neurons [27,28].

Our data suggest that bicuculline and muscimol alter the maturation of the dendritic morphology of motoneurons by inhibiting the generation of network activity in the chicken spinal cord. Two main findings support this conclusion. First, exposure of isolated spinal cords to bicuculline or muscimol inhibits the generation of network activity *in vitro *under our experimental conditions. The inhibitory effect of bicuculline and muscimol on network activity is most likely caused by blocking the activation of endogenous GABA_A _receptors and/or shifting the chloride equilibrium potentials in the motoneurons required for the generation of spontaneous episodes [3,7,9]. In this regard, it is interesting to point out that most of the spontaneous activity-evoked changes in intracellular Cl^- ^driven by GABA receptor activation occur in the dendrites, rather than in the cell body of the motoneurons [9]. Disruption of GABA-driven network activity by bicuculline and muscimol could explain how these two treatments, which have opposite outcomes on the activation of endogenous GABA_A _receptors, generate similar effects on the dendritic outgrowth of the motoneurons. However, it is unclear whether treatment of chicken embryos with bicuculline and muscimol generates similar temporal patterns of inhibition of spontaneous network activity *in ovo*. It is very likely that some differences may exist in the temporal pattern of inhibition of spontaneous activity following *in ovo *application of bicuculline and muscimol [6,29]. For example, previous findings suggest that *in ovo *application of bicuculline only inhibits the generation of spontaneous activity for a short period of time (< 12 hours) since blockade of GABA receptor activation triggers a compensatory mechanism that restores network function [29]. Nonetheless, the combined effect of daily application of either drug for 3 to 4 days (even if it only results in a transient disruption of network activity) is sufficient to inhibit the normal pattern of dendritic outgrowth in developing motoneurons. Second, the idea that GABA-driven activity regulates the maturation of dendritic outgrowth in the motoneurons is also supported by the effect of Kir2.1 on dendritic morphology. We have previously demonstrated that overexpression of Kir2.1 in the chicken spinal cord disrupts the electrical excitability of motoneurons by decreasing the resting membrane potential and input resistance. This will ultimately decrease the frequency of spontaneous motor activity in chicken embryos [2]. Our data show that disruption of network function with the Kir2.1 inward rectifier K^+ ^channel inhibits the normal dendritic development at E10. Furthermore, the inhibitory effect of Kir2.1 expression on dendritic outgrowth of E10 motoneurons was comparable to that evoked by *in ovo *treatment of chicken embryos with bicuculline or muscimol between E8 and E10.

The inhibitory effect of bicuculline and muscimol on the maturation of the dendritic morphology was unlikely due to a toxic effect on the motoneurons. Thus, treatment of chicken embryos with bicuculline at all ages tested had no effect on motoneuron survival, whereas muscimol treatment of chicken embryos *in ovo *caused a significant increase in the number of Islet1/2-positive neurons in the spinal cord. There have been conflicting reports regarding the effect of muscimol on motoneuron survival in the chicken spinal cord [23,30]. Our results support the findings of Oppenheim *et al*. [30], who demonstrated that treatment of chicken embryos with a similar dose of muscimol causes a significant increase in motoneuron survival in the lumbar spinal cord. It was concluded that the stimulatory effect of muscimol on motoneuron survival was due primarily to increased nerve branching and access to target-derived factors generated by inhibition of neuromuscular activity [30]. If this is the case, it is puzzling that bicuculline treatment of chicken embryos has no significant effect on motoneuron survival. Our experiments demonstrate that bicuculline treatment of the isolated spinal cord preparation causes a complete inhibition of network activity at all ages tested. Two factors may have contributed to the differential effect of muscimol and bicuculline on motoneuron survival. First, there may be temporal differences in the actions of bicuculline and muscimol *in ovo *(that is, how long they remain effective before being eliminated). Second, the depolarizing effect of muscimol, which results in a significant influx of intracellular calcium [2], may also influence motoneuron survival [31].

Disruption of the normal pattern of dendritic development by inhibiting GABA-driven activity has no effect on the maturation of spontaneous electrical activity generated by spinal cord networks at early stages of development (E6 to E8). A similar effect was also observed at later stages of development (E8 to E10) following inhibition of GABA-driven activity by muscimol but not bicuculline. It is believed that dendritic morphology regulates the ability of developing neurons to establish appropriate synaptic contacts with other network components and to integrate synaptic inputs, which can have a dramatic effect on the formation and function of neuronal circuits [32]. Surprisingly, our results show that chronic disruption of GABAergic neurotransmission (and the resulting inhibition of dendritic outgrowth) did not alter the ability of spinal cord networks to generate episodes of spontaneous activity. Thus, chronic treatment of chicken embryos with bicuculline (between E6 and E8) or muscimol (between E6 and E8 or between E8 and E10) has no effect on the shape or the duration of the episodes of spontaneous activity recorded at E8 and E10. Therefore, it appears that the maturation of spinal cord network activity is not affected by disrupting the dendritic outgrowth of the motoneurons. This may occur because disruption of GABA receptor activation leads to homeostatic changes in the cellular excitability and synaptic strength of spinal motoneurons [29,33,34]. For example, inhibition of GABA receptor activation in the chicken embryo triggers an increase in Na^+ ^currents and a concomitant reduction in the expression of K^+ ^conductances, resulting in an overall increase in cellular excitability [29]. Inhibition of GABA receptor activation is also followed by a significant increase in the synaptic strength of excitatory GABA and glutamate synaptic potentials [34]. These changes in cellular excitability and synaptic transmission may contribute to the recovery of spontaneous activity in depressed spinal cord networks (following inhibition of GABA-driven activity). Thus, it appears that inhibition of dendritic outgrowth by disruption of GABA receptor activation triggers compensatory changes in network connectivity in order to maintain appropriate levels of activity in the motoneuron pool. In this context, our results suggest that changes in the intrinsic properties of the motoneurons and their connectivity with other network components have the ability to compensate for changes in dendritic morphology evoked by disruption of GABA receptor activation in the chicken spinal cord. Although disruption of GABA receptor activation has no effect on the shape and duration of the episodes of spontaneous activity, we recorded a significant increase in the inter-episode interval when chicken embryos were treated with bicuculline between E8 and E10 but not with muscimol treatment during this developmental period. The inter-episode interval produced during normal spontaneous activity is generated by the interplay of the initial triggering event from the network generator into the motoneurons and the presence of a slow form of activity-dependent network depression, whereas the episode duration is regulated by the duration of the previous inter-episode interval [35-37]. Accordingly, it appears that only the bicuculline-induced reduction in dendritic outgrowth may interfere with the ability of motoneurons to connect to specific network components. Muscimol-evoked reduction in dendritic outgrowth may not result in similar changes in network connectivity because of the increased number of motoneurons detected with our Islet staining.

GABA-driven network activity could potentially regulate the maturation of dendritic morphology in a direct or indirect manner. We have previously demonstrated that GABA receptor activation in motoneurons leads to a considerable increase in intracellular calcium in the soma of the motoneurons [2]. This stimulatory effect of GABA on intracellular calcium can potentially regulate dendritic outgrowth, as has been demonstrated in cerebellar granular cells [38,39]. Changes in intracellular Ca^2+ ^evoked by activation of GABA_A _receptors regulates the stability of dendritic growth cones and promotes dendritic elongation in developing neurons [38]. GABA-driven network activity could also regulate dendritic morphology indirectly by regulating the release of neurotrophic factors like brain-derived neurotrophic factor (BDNF) [40,41]. Previous findings have uncovered an important role for activity-dependent BDNF release in regulating the maturation of dendritic morphology in developing neurons [42,43]. Future experiments will establish the precise molecular and cellular mechanisms used by GABA-driven activity in regulating the maturation of dendritic morphology of spinal motoneurons *in vivo*.

## Abbreviations

E: embryonic day; GABA: gamma-aminobutyric acid; PBS: phosphate buffered saline.

## Competing interests

The authors declare that they have no competing interests.

## Authors' contributions

YJY and MMC conceived and designed the experiments. YJY performed the analysis of dendritic outgrowth and cell survival. APG performed the recordings of network activity. YJY, APG and MMC analyzed the data. YJY and MMC wrote the paper. All authors read and approved the final manuscript.
